# Deregulation of All-*Trans* Retinoic Acid Signaling and Development in Cancer

**DOI:** 10.3390/ijms241512089

**Published:** 2023-07-28

**Authors:** Geoffrey Brown

**Affiliations:** School of Biomedical Sciences, Institute of Clinical Sciences, College of Medical and Dental Sciences, University of Birmingham, Edgbaston, Birmingham B15 2TT, UK; g.brown@bham.ac.uk; Tel.: +44-(0)121-414-4082

**Keywords:** retinoid metabolism, RARs, cell development, stem cells, cancer, oncogenes

## Abstract

Cancer stem cells are the root cause of cancer, which, in essence, is a developmental disorder. All-*trans* retinoic acid (ATRA) signaling via ligand-activation of the retinoic acid receptors (RARs) plays a crucial role in tissue patterning and development during mammalian embryogenesis. In adults, active RARγ maintains the pool of hematopoietic stem cells, whereas active RARα drives myeloid cell differentiation. Various findings have revealed that ATRA signaling is deregulated in many cancers. The enzymes for ATRA synthesis are downregulated in colorectal, gastric, lung, and oropharyngeal cancers. ATRA levels within breast, ovarian, pancreatic, prostate, and renal cancer cells were lower than within their normal counterpart cells. The importance is that 0.24 nM ATRA activates RARγ (for stem cell stemness), whereas 100 times more is required to activate RARα (for differentiation). Moreover, RARγ is an oncogene regarding overexpression within colorectal, cholangiocarcinoma, hepatocellular, ovarian, pancreatic, and renal cancer cells. The microRNA (miR) 30a-5p downregulates expression of RARγ, and miR-30a/miR-30a-5p is a tumor suppressor for breast, colorectal, gastric, hepatocellular, lung, oropharyngeal, ovarian, pancreatic, prostate, and renal cancer. These complementary findings support the view that perturbations to ATRA signaling play a role in driving the abnormal behavior of cancer stem cells. Targeting ATRA synthesis and RARγ has provided promising approaches to eliminating cancer stem cells because such agents have been shown to drive cell death.

## 1. Introduction

Gradients of ATRA from embryonic regions that synthesize together with juxtaposed local degradation play crucial roles in the precise patterning of mammalian embryonic development. Similarly, vitamin A deficiency within mammalian and chicken embryos has led to tissue disorganization and multiple defects [1,2]. The three main types of RARs for ATRA, namely RARα, RARβ, and RARγ, are transcriptional regulators, whereby ATRA activates obligate dimers between one of the RARs and one of the retinoid X receptors (RXR)—α, β, or γ. ATRA binding results in gene activation, and expression is repressed in the absence of ATRA due to a lack of displacement of corepressors and the recruitment of coactivators [3]. Orchestration of ATRA levels together with the combined actions of different RAR/RXR dimers ensures that embryonic patterns arise in a spatial and temporal manner via regulating the expression of genes for cell survival/proliferation and differentiation, including lineage specification.

Deregulated RAR signaling occurs in cancer, whereby acute promyelocytic leukemia (AML-M3) provides a paradigm. The cardinal reciprocal translocation t(15;17) (q24;q21) involves the *PML* and *RARA* genes [4], and there are few other cytogenetic defects. The leukemia cells resemble promyelocytes and, therefore, an assumption was that the leukemia arises in a committed myeloid progenitor cell. However, a case was made against this origin and to favor a target cell that had not undergone lineage restriction [5]. The evidence to support the view that acute promyelocytic leukemia arises from transformation of a hematopoietic stem cell, rather than a committed myeloid progenitor, is that the t(15;17) (q24;q21) translocation and the oncogenic fusion protein are both present in patients’ hematopoietic stem cell-like CD34+ CD38− cells. In this case, there is then restriction of the leukemia stem cells to promyeloid development [6]. ATRA targeting of the oncogenic PML–RARα fusion protein, together with the use of arsenic trioxide, has provided a cure for most patients with acute promyelocytic leukemia [4,7], but ATRA has failed to provide an effective treatment for other cancers [8,9,10,11]. Rearrangements of the *RARG* gene, rather than of the *RARA* gene, have been identified in acute promyelocytic leukemia patients and involved the *NUP98* (for nucleoporin, 3 patients), *CPSF6* (encodes an RNA-binding protein, 4 patients), *NPM1* (for nucleophosmin, 1 patient), and *PML* (1 patient) genes. The patients failed to respond to ATRA-based therapy other than the one with the *PML–RARG* translocation [12].

As for acute promyelocytic leukemia, a principle to categorizing the leukemias, and other cancers, is the affiliation of malignant cells to a cell lineage. A sub-population of cells, termed cancer stem cells, sustain the hierarchy of cells for cancer [13]. Cancer stem cells may arise for many cancers from the transformation of a tissue-specific stem cell, but the development of cancer stem cells is abnormal because their offspring is often restricted to a developmental pathway. Some cancers may arise from transformation of a committed/lineage-affiliated cell, but the malignant cells still do not behave as normal primitive progenitors. From recent findings for hematopoiesis, hematopoietic stem cells can directly acquire a lineage bias/affiliation to a lineage pathway from a continuum of all options. However, their subsequent development trajectory is both broad and versatile. The boundaries between trajectories are mutable because affiliated stem/progenitor cells are still able to adopt a different and closely related pathway (reviewed in [14]). In this case, the developmental trajectory of cancer stem cells that might have arisen from a committed progenitor is also constrained. When some oncogenes were targeted to hematopoietic stem/progenitor cells, the offspring of the leukemia stem cells belonged to a cell lineage, providing further support to programming of cancer stem cells to a cell lineage [15]. Finally, cell differentiation is impaired for some cancer cells, and we might, therefore, expect retinoid signaling to be disrupted.

This review outlines the extent to which the RAR-mediated controls on developmental processes are deregulated in cancer cells. Known perturbations include the expression of key enzymes for ATRA biosynthesis, the intracellular levels of ATRA, the balance of expression of RARs and, particularly, RARγ overexpression, and the levels of miR-30a-5p, which is a negative regulator of RARγ and a tumor suppressor gene. Collectively, the findings support the view that perturbations to RAR signaling play a role in the abnormal development of cancer stem cells.

## 2. ATRA Synthesis within Normal and Cancer Cells

Studies that have examined the impact of ATRA signaling on cancer cells have mostly treated cultured cells with 1 to 5 mM, a pharmacological amount, to see whether they differentiate. Contrary to this approach, and germane to the pathophysiology of a patient’s cancer, is whether cancer cells can synthesize ATRA from all-*trans* retinol for the activation of RARs, and whether there is constitutive disruption to ATRA-driven RAR-mediated events. Cells synthesize ATRA from all-*trans* retinol (dietary vitamin A) that is present in 10% fetal calf serum-supplemented medium, which contains 50 nM all-*trans* retinol and 0.4–1.4 nM ATRA [16].

The first step in ATRA biosynthesis is all-*trans* retinol oxidation to all-*trans* retinaldehyde (retinal). The enzymes of physiological importance are still unclear, other than they are the cytosolic alcohol dehydrogenases (ADHs) and the microsomal NAD+-dependent retinol dehydrogenases (RDHs). RDH1, RDH10, and DHRS9 are viewed as first-step physiological catalysts [17], but other enzymes are of interest. NADP+-dependent RDHs and cytosolic aldo–keto reductases (AKRs) convert all-*trans* retinaldehyde back to retinol. Irreversible oxidation of all-*trans* retinaldehyde to ATRA is catalyzed by the cytosolic aldehyde dehydrogenases (ALDH) ALDH1A1 (also known as RALDH1), ALDH1A2 (RALDH2), and ALDH1A3 (RALDH3) [17,18]. There is reduced ATRA synthesis within some cancer cells, as shown by a lack of the required enzymes, a low level of conversion of all-*trans* retinol to ATRA, and reduced levels of ATRA within cancer cells as compared to their normal counterparts. 

### 2.1. Colorectal Cancer

The expression of multiple genes that are involved in ATRA synthesis is altered in colorectal cancer as compared with normal colorectal tissue. From a literature analysis of microarray, RNA-Seq, and EST databases, oxidation of all-*trans* retinol to all-*trans* retinaldehyde within normal colorectal tissue was associated with expression of mRNAs for ADH1B, ADH1C, and ADH3, and RDHL mRNA was detected at a lower abundance by RNA-Seq. Significant downregulation of ADH1B, ADH1C, and RDHL mRNAs was seen for colorectal cancer samples. mRNAs that encoded the all-*trans* retinaldehyde-reducing enzymes RDH11 and AKR1B10 were present in normal colorectal cells, and AKR1B10 mRNA was the most abundant and reduced in colorectal cancer samples. Expression of the above enzymes was examined by semi-quantitative RT-PCR for paired colorectal cancer and normal tissue samples from 30 patients. ADH1B and ADH1C mRNAs were present at very high levels in normal colon and rectal tissues; RDHL, RDH5, and RDH10 mRNAs were at lower levels. ADH1B, ADH1C, RDHL, and RDH5 mRNAs were decreased in colorectal cancer samples, and expression of ADH1B and ADH1C mRNAs decreased regarding progression from adenoma to early and more advanced colorectal cancer. Regarding the oxidation of all-trans retinaldehyde to ATRA, ALDH1A1 mRNA was expressed predominantly in the large intestine. A decrease was observed for 60% of colorectal samples, but the reduction was not significant. A similar pattern of changes was observed for the colorectal cancer cell lines HCT-116, HT-29, and RKO [19]. 

Comparison of benign colon adenomas, colon cancer, and normal colon tissue by microarray and RT-PCR analyses revealed reduced expression of the genes *RDHL* and *RDH5* in adenoma and cancer samples. The colon cancer cell lines Caco-2, Colo205, DLD-1, HCT116, HT29, RKO, and SW480 lacked expression of these enzymes and showed poor conversion of all-*trans* retinol into ATRA as compared with normal human mammary epithelial cells. Adenomatous polyposis gene mutations contributed to the cause of colon adenoma and cancer regarding the control of colonocyte proliferation. The investigators provided evidence to support the view that the adenomatous polyposis gene and the gene that encodes the colon-specific factor CDX2 play roles in regulating the expression of *RDHL* and ATRA synthesis [20]. From the above, the impairment to ATRA synthesis in colorectal cancer appears to contribute to disease progression. 

### 2.2. Gastric Cancer

Analysis of transcriptome databases and semi-quantitative RT-PCR were undertaken to compare human malignant and normal gastric tissues regarding the levels of enzymes for ATRA biosynthesis. Reduced mRNAs in most tumor samples included ADH4, ADHIB, ADHIC, and RDHL (for oxidation of all-*trans* retinol to all-*trans* retinaldehyde), and AKR1B10, AKR1B1, and RDH12 (for reduction of all-*trans* retinaldehyde to all-*trans* retinol). A significant decrease in the level of ALDH1A1 mRNA (oxidation of all-*trans* retinaldehyde to ATRA) was observed for malignant gastric cancer tissue. From these findings, the investigators concluded that the changes could lead to a significant reduction in the level of ATRA within gastric cancer tissue [21]. Other investigators have reported that ADH4 activity correlated with ATRA synthesis in the human gastric mucosa and that AHH4 activity and ATRA synthesis decreased with increasing levels of inflammation, atrophy, and intestinal metaplasia. They concluded that ATRA production is important to the maintenance of normal morphology, and a reduction may be involved in the pathogenesis of gastric mucosa [22].

### 2.3. Lung Cancer

From RT-PCR studies, significant decreases in the levels of mRNAs for ADH1B, ADH3, RDH1, and ALDH1A1 were observed within 82%, 79%, 73%, and 64% of small-cell lung cancer samples, respectively, as compared to normal tissue. Decreases were observed at the early stages of malignant transformation and the investigators concluded that disruption to ATRA biosynthesis is a feature of lung tissue transformation. The level of mRNA for AKR1B10 (reduces all-*trans* retinaldehyde to retinol) was increased within 80% of tumor samples [23].

### 2.4. Oropharyngeal

From immunohistochemical analyses of primary oropharyngeal squamous cell cancer, a low level of expression of ALDH1A2 was associated with a mesenchymal-like phenotype (vimentin expression) and an unfavorable prognosis. For patients with high ALDHIA2, a favorable prognosis correlated with high expression of cellular retinoic acid-binding protein 2 (CRABP2). Xenograft FaDu cell tumors with stable silencing of ALDH1A2 expression were mesenchymal-like and exhibited accelerated growth, and treatment of FaDu cells with the ALDH1A2 inhibitors WIN18.446 or BMS493 led to a loss of cell adhesion and a mesenchymal-like phenotype [24]. Previously, *ALDH1A2* gene promoter hypermethylation has served as a risk factor for an unfavorable prognosis [25].

We might expect ATRA synthesis to be compromised in cancer cells from the above findings. However, a caveat is the need to pay regard to the developmental status of the cancer versus normal cells examined, and whether the expression of an enzyme (or enzymes) that is (are) of primary importance to ATRA synthesis at a particular stage in cell development has been examined. It has been shown that the levels of expression of ALDH1A1, ALDH1A2, and ALDH1A3 change during rat nephron development [26]. Hence, a significant decrease in the level of expression of a particular enzyme within cancer cells may or may not reflect a low capacity to synthesize ATRA. Even so, further evidence to support a low level of ATRA within cancer cells has been provided by studies that have exposed cancer cells and their normal counterparts to all-*trans* retinol and that have measured ATRA levels within matched tissues.

### 2.5. Breast Cancer

Normal human mammary epithelial cells produced 5 and 10 times more ATRA from all-*trans* retinol than the human breast cancer cell lines MCF-7 and MDA-MB-231, respectively. A decrease in all-*trans* retinol within normal human mammary epithelial cells was also attributed to the rapid formation of retinyl esters. In keeping with the increased ATRA production by normal human mammary epithelial cells, they were markedly more sensitive to growth inhibition by a physiological dose of retinol (2.5 mM) than the breast cancer cell line MCF-7, and MDA-MB-231 cells were not affected [27]. Other workers have reported that normal human mammary epithelial cells and the non-tumorigenic breast cell lines synthesized ATRA, whereas five of six breast cancer cell lines either did not synthesize ATRA or did so at a low rate. Retinol (at 0.5 to 2 mM) inhibited the growth of the non-tumorigenic MCF-10F cell line but not those of the MCF-7 and T47D cell lines. A strong induction of the CYP26A1 (ATRA metabolizing) did not correlate with the lack of ATRA in breast cancer cells because such was confined to the ERα-positive MCF-7 and T47D cell lines [28].

### 2.6. Ovarian Cancer 

Cultures of four immortalized human ovarian epithelial cells lines produced ATRA when exposed to all-*trans* retinol, and the conversion was efficient because little intracellular all-*trans* retinol was detected. None of the ovarian cancer cell lines OVCAR3, OVCAR10, A1847, A2780, and SKOV3 produced detectable ATRA as the cells lacked ALDH1H2, leading to a complete loss of all-*trans* retinaldehyde oxidation. One of the two SV40-immortalized human ovarian epithelial cell lines made ATRA [29]. Other investigators have reported, for the ovarian cancer cell lines YDOV-139, YDOV-157, YDOV-161, YDOV-13, YDOV-105, and YDOV-151, as compared with normal human ovarian epithelial cells, that the *ALDH1A2* gene was the most prominent downregulated regarding *ALDH* family members. *ALDH1B1* and *ALDH9A1* gene expression were also downregulated in the ovarian cancer cells, and *ALDH3A1* expression was upregulated. When ovarian cancer cell lines were compared to immortalized human ovarian surface epithelial cells, hypermethylation of the *ALDH1A2* gene was higher in the cancer cell lines [30].

### 2.7. Pancreatic Cancer 

The levels of retinoids within tissues from patients with pancreatic ductal adenocarcinoma and murine tumors from the orthotopic PanC02 model were measured by high-performance liquid chromatography mass spectroscopy. ATRA and all-*trans* retinol levels were reduced when the human tumor tissues were compared with healthy pancreatic tissue. Only the concentration of ATRA was reduced when the mouse tissues were compared. The expression of *ALDH1A1* was reduced in human and mouse tumor tissues compared to a normal pancreas. Real-time RT-PCR examination of the expression of RARs revealed downregulation of RARα, RARβ, RXRα, and RXRβ in pancreatic ductal adenocarcinoma tissue. An improved patient survival was associated with expression of RARα, RXRβ, and lecithin–retinol acyltransferase, which converts all-*trans* retinol into retinyl esters for storage [31]. Other workers have reported a lack of ATRA signaling for chemically induced and genetically engineered mouse models of pancreatic cancer, concluding that it plays a role in tumorigenesis [32]. 

### 2.8. Prostate Cancer 

High-performance liquid chromatography analysis was undertaken to determine the levels of retinoids within prostate cancer, benign prostate hyperplasia, and normal prostate tissue samples. Prostate cancer tissue contained 5 to 8 times less ATRA than benign prostate hyperplasia, and normal prostate and the level in prostate cancer tissue was at or near to the limit of detection (at 1 ng/g wet wt.). The level of all-*trans* retinol within prostate cancer tissue was near normal (77 ng/g wet wt. for cancer tissue and 60 ng/gr wet wt. for normal tissue), and the all-*trans* retinol concentration in benign prostate epithelium was 2.5 times that of the other two tissues. Metabolic studies limited to benign prostate hyperplasia showed that the enzyme activities that are required to convert all-*trans* retinol to ATRA, via all-*trans* retinaldehyde, were present. RARα, RARβ, and RARγ mRNAs were expressed by normal and tumor samples [33]. 

### 2.9. Renal Cell Carcinoma

From studies using radiolabeled all-*trans* retinol, the levels of all-*trans* retinol and retinyl esters for ATRA production were greatly reduced in renal cell cancers as compared to a normal kidney [34]. Lecithin–retinol acyltransferase (LRAT), which converts all-*trans* retinol to retinyl esters, was detected, by immunohistochemical analysis, at high levels in tubule epithelial cells and the Bowman’s capsule lining in the glomeruli of normal kidney. Despite reduced levels of retinyl esters in renal tumors, LRAT expression was higher in renal tumors than in normal kidneys, particularly high in tumors that were indolent, and seemed to be a marker of renal tumors that were benign or of low malignant potential [35].

The reduced level of ATRA seen for some cancers is important to pathophysiology for two reasons. RARγ is transactivated by an exceedingly low concentration of ATRA (0.24 nM), and 100 times more is required to transactivate RARα (19.3 nM) [36,37]. Hence, there should be differential usage of RARγ within cancer cells that have or are expected to have a low intracellular level of ATRA. Furthermore, RARγ is an oncogene for some of the cancers that have a low intracellular level of ATRA.

## 3. RARγ Is Overexpressed by Some Carcinomas

RARγ is overexpressed in human colorectal cancer [38], cholangiocarcinoma [39], hepatocellular cancer [40], ovarian cancer [41], pancreatic ductal adenocarcinoma [42,43], and renal cell cancer [44]. High levels of expression were associated with increased cell proliferation, rapid tumor progression, and a poor prognosis. Overexpression of RARγ within hepatocellular cancer cell line cells promoted colony formation and the growth of xenografts in nude mice [40]. High-level RARγ expression within ovarian cancer cells was related to FIGO stages III–IV and a survival time of <5 years [41]. For cholangiocarcinoma, a high level of RARγ expression and a poor prognosis were related to resistance to 5-fluorouracil [39]. Pancreatic ductal adenocarcinoma is often diagnosed at an advanced stage and is one of the most lethal malignancies. Investigators have reported that RARγ overexpression was increased during tumor progression and correlated with a significantly worse prognosis [43].

Findings from studies of RARγ knockdown and knockout within cancer cells have provided support to a role in promoting cell proliferation. Knockdown of RARγ overexpression within human colorectal cancer cell lines increased sensitivity to 5-fluorouracil, oxaliplatin, and vincristine by reducing the expression of multidrug resistance protein 1 [38]. Si-RNA knockdown of RARγ expression suppressed the xenograft growth of the cholangiocarcinoma cell line QBC939 [39]. RARγ was required for the proliferation of pancreatic ductal adenocarcinoma cells as CRISP-9-Cas9 knockout in PANC1 cells led to cycle arrest at S phase and apoptosis, and tumor formation by *RARG*-deficient cells in NOD/SCID mice was suppressed. As opposed to binding to RAREs, RARγ binding to the *MYC*, *STAT3*, and *SLC2A1* gene promoters seemed to mediate chromatin epigenetic activation regarding deposition of the activation marker histone H3 K27 acetylation [42]. Si-RNA knockdown of expression suppressed the proliferation of patient-derived pancreatic ductal adenocarcinoma organoids and pancreatic cancer cell lines. Contrary to the above findings, blocking RARγ signaling within Panc-1 and PK-1 cells upregulated p21 and p27, and the cells arrested in G1 of cell cycle but did not undergo apoptosis [43]. Intriguingly, downregulation of the expression of RARα and RARβ has been reported in pancreatic ductal adenocarcinoma and associated with better overall survival [31].

RARγ plays a role in modulating the level of ATRA within cells by regulating the expression of genes that encode regulators of ATRA metabolism and signaling. The genes, as revealed by comparison of ATRA-treated will type and RARγ null embryonic stem cells, were those that encoded stimulated by retinoic acid 6 (STRA6), LRAT, CRABP2, and CYP26A1 [45]. These proteins transfer all-*trans* retinol from the blood (as bound to retinol-binding protein 4 (RBP4)) into cells, convert all-*trans* retinol into retinyl esters for storage, deliver ATRA to the nucleus and RARs, and catabolize ATRA to polar metabolites, respectively. From zebrafish studies, CYP26A1 was seen to be robustly regulated by ATRA, even when the availability was reduced [46]. A sustained low level of ATRA within stem cells, via RARγ upregulation of expression of CYP26A1, may protect cells from differentiating from activation of RARα, which requires a high level of ATRA.

## 4. MiR Regulation of Retinoic Acid Signaling 

The miR-30 family members are of particular interest for various reasons. The family contains the five pre-mature miR-30a, miR-30b, miR-30c, MiR-30d, and miR30-e, and the 3p and 5p miR-30s are processed from the same precursor but from different regions. miR-30s are regulators of tissue and organ development and clinical diseases (reviewed in [47]). They target different genes and pathways to perform various roles with family members sharing a regulatory network. The finding that supported a shared network was that, whilst family members were located at three different chromosomal regions, their patterns of expression were similar for different multiple myeloma samples regardless of copy number alterations. For multiple myeloma, miR-30s is a tumor suppressor that targets the constitutively active Wnt/β-catenin pathway [48], a pathway that is important to stem cell renewal during embryogenesis and within adult tissues. As follows, miR-30a is a tumor suppressor for many cancers and integrates ATRA biosynthesis and RARγ expression.

### 4.1. MiR-30a Regulates ATRA Biosynthesis 

ALDH1A2 is a rate-limiting enzyme in the synthesis of ATRA. As mentioned above, histological studies of oral squamous cancer revealed that low level expression of ALDH1A2 was linked to an unfavorable outcome. Other works have shown, for matched oral squamous cell cancer and normal tissue samples, that the *ALDH1A2* and *ADHFE1* genes were frequently hypermethylated, and the levels of mRNAs for ADHFE1 and ALDH1A2 were significantly downregulated. In this study, miR-30a and mR-379 were reported as positive regulators of *ALDH1A2* and *ADHFE1* gene expression, via targeting the DNA methyltransferase 3B. This enzyme was differentially expressed in tumor samples and correlated significantly with *ALDH1A2* and *ADHFE1* gene expression. Ectopic expression of miR-30a and miR-379 within the SCC-15 and OEC-M1 cell lines led to re-expression of methylation-silenced *ALDH1A2* and *ADHFE1*, the colony-forming ability of transfected cells was reduced, and cell growth was inhibited [49]. In addition to miR-30a modulation of ATRA biosynthesis, it has been reported that ATRA modulates the expression of miR-30a. Treatment of the gastric cancer AGS and MKN-45 cell line cells with cisplatin led to downregulation of miR-30a mRNA. Pre-incubation of these cells with ATRA increased miR-30a mRNA expression which enhanced the sensitivity of cells to growth inhibition by cisplatin and its cytotoxicity [50]. 

### 4.2. MiR-30a-5p Negatively Regulates RARγ Expression and Is a Tumor Suppressor for Various Cancers

MiR-30a-5p negatively regulates the expression of RARγ, as transfection of human immortalized cord-blood stem cells with miR-30a-5p diminished *RARG* mRNA and RARγ levels [51]. MiR-30a is a known tumor suppressor for breast, colorectal, gastric, hepatocellular, lung, oropharyngeal, ovarian, pancreatic, prostate, and renal cancers. MiR-30a expression was widely downregulated in patients’ colorectal, gastric, hepatocellular, lung, oral, pancreatic, prostate, and renal cancer tissues, and accordingly colorectal, hepatocellular, pancreatic, and renal cancer cells are known to overexpress RARγ. 

MiR-30a levels were low in colorectal cancer patients’ cells, and transfection suppressed the proliferation and colony-forming efficiency of the colorectal cancer cell lines HCT116 and SW620, as well as the growth of HCT116 cells, in a mouse xenograft model. The suppressive effect of MiR-30a was attributed to targeting of heterochromatin protein 1g expression, which promotes colon cancer progression, and downregulates p21 expression via methylation of its promoter. The investigators proposed that the miR-30a/heterochromatin protein 1g/p21 axis controls colon cancer development [52]. Lower levels of miR-30a-5p in gastric cancer patients’ cells were linked with distant metastases and a reduction of progression-free survival rates [53]. MiR-30a-5p expression was lower in hepatocellular cancer tissues (and low in cell lines) compared to adjacent non-cancerous tissue, and ectopic expression of miR-30a-5p inhibited cell proliferation [54]. The miR-30s are expressed in adult and mouse lungs; there was reduced expression in lung cancer, and they have been described as gate keepers in lung cancer [55]. A low level of expression of miR-30a-5p in oral cancer tissues was linked to the proliferation and invasion of oral cancer cells [56]. The levels of expression of miR-30a, miR-30b, and miR-30c were lower in pancreatic ductal adenocarcinoma tissues compared to a normal pancreas. Enforced expression of miR-30a and miR30b led to suppression of the growth in vitro and in vivo of the pancreatic ductal adenocarcinoma MiaPaCa-2 and Panc-1 cells, and the miR-30 gene was hypermethylated in MiaPaCa-2 pancreatic cancer cells [57]. MiR-30a-5p was downregulated in prostate cancer tissue, and long non-coding RNA activated by DNA damage (NORAD) was upregulated, while NORAD binds to and downregulates miR-30a-5p. Overexpression of NORAD within PC-3 cells promoted proliferation and trans-well invasiveness, and the additional overexpression of miR-30a-5p attenuated the NORAD-mediated promoting effects [58]. Decreased expression of miR-30a-5p in renal cell cancer was correlated with a higher clinical grading of cancer tissues, and overexpression of miR-30a-5p inhibited the growth of and colony formation by the renal cell cancer cell lines 786-0 and OS-RC-2 [59]. Downregulation of miR-30a-5p expression renal cell cancer was associated with gene promoter methylation and a shorter time to relapse, whereby miR-30a-5p^me^ has provided a diagnostic and prognostic biomarker [60].

Overexpression of miR-30s in breast and ovarian cancer cells inhibited their proliferation. Overexpression of miR-30a-5p within the breast cancer MCF-7 and ZR75-1 cell lines reduced cell proliferation and colony formation. The transcription cofactor Eya2 was upregulated in breast cancer patients and miR-30a-5p suppression was attributed to targeting of Eya2 [61]. Growth suppression of breast cancer cells by MiR-30a-5p has also been attributed to dampening of aerobic glycolysis by inhibition of lactate dehydrogenase A and expression of this enzyme correlated negatively with expression of miR-30a-5p for breast cancer patients [62]. Other workers have reported miR-30c-2-3p was consistently upregulated in estrogen receptor positive breast cancer and suggested that family members may place dualistic roles in cancer [63]. Overexpression of miR-30 in the human ovarian SK-OV-3 and A2780 cancer cell lines inhibited their proliferation and invasiveness, and overexpression of RAB32, a member of the RAS oncogene family, prevented miR-30 tumor suppression [64]. Methylation of the promoter region of the *miR-30* gene has been reported for oropharyngeal squamous cell cancer, and xenograft growth of the squamous cell cancer lines UM-SCC-46 and UM-SCC-47 was inhibited by a miR-30a-5p mimic formulated into a targeted nanomedicine [65].

MiR-30-5p is commonly at a low level within cancer cells and a tumor suppressor. By contrast, miR-30a-5p expression was increased in cholangiocarcinoma tissues. Inhibition of miR-30a-5p in the REB cell line (high miR-30a-5p) inhibited the proliferation of these cells, and overexpression in the HCC9810 cell line (low miR-30a-5p) promoted proliferation. MiR-30a-5p also promoted the tumorigenicity of cholangiocarcinoma cells from studies using a xenograft model [66].

## 5. ATRA Signaling in Cancer

**Figure 1:**
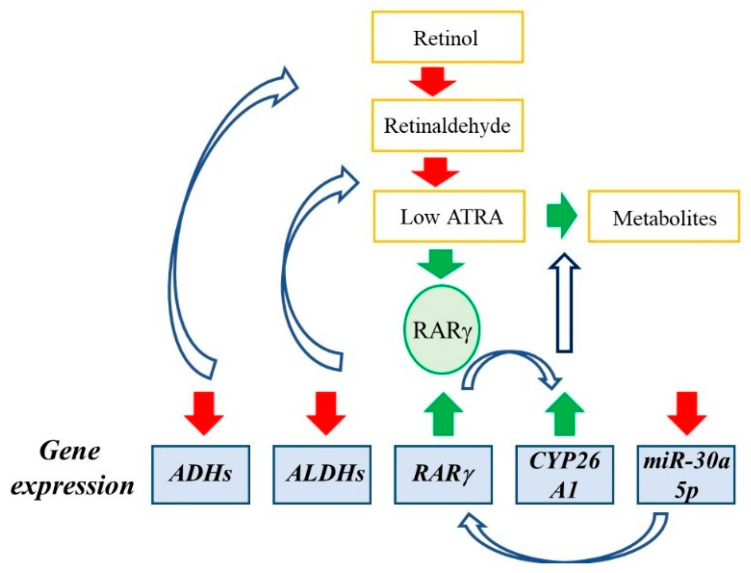
Perturbations to ATRA synthesis and signaling in cancer cells. For some cancers, there is reduced expression of the oxidizing enzymes to the synthesis of ATRA (shown by the red downward arrows), leading to reduced ATRA synthesis (shown by the open curved arrows). Some cancers have a low intracellular level of ATRA and overexpress RARγ (green upward arrow) which is activated by nM levels of ATRA. RARγ positively regulates the expression of CYP26A1 (shown by the open curved arrows) to contribute to a low intracellular level of ATRA via metabolism of ATRA. For some cancers, the level of miR30a-5p is low (red downward arrow) and miR-30a-5p is a tumor suppressor. It downregulates expression of RARγ (shown by the open curved arrow) and positively regulates the expression of ALDH1A2 and ADHFE1. For the perturbations within different cancers, see the text.

Table 1 summarizes the key findings for ATRA signaling in various cancers. Findings to support reduced ATRA synthesis include a lack of the enzymes that are involved and measurements of the conversion of all-*trans* retinol to ATRA and of the intracellular concentration of ATRA. RARγ is activated by nM levels of ATRA and an oncogene by virtue of overexpression which promotes tumor growth. miR30a-5p is low in patients’ cancer cells, a tumor suppressor, and downregulates expression of RARγ. The findings are complementary regarding disruption to ATRA signaling in cancer cells other than for unknown reasons RARγ is overexpressed by cholangiocarcinoma cells and miR-30a-5p is a tumor promoter. Figure 1 depicts the interrelationships between disruption to ATRA synthesis and expression of RARγ and miR-30a-5p. The above measurements using bulk cancer cells provide a picture of events within the whole population of cells.

Impaired ATRA synthesis coupled to overexpression of RARγ within cancer cells are features that have been attributed to normal stem cells. Mouse pluripotent embryonic stem cells appear to lack the machinery to synthesize ATRA because of an absence of ADH1, ADH4, and RALDH2 and STRA6. This study reported a growth factor-like function for all-*trans* retinol via activation of phosphoinositide 3-kinase [67,68]. RARγ expression is restricted to hematopoietic stem cells and primitive progenitors and, therefore, a feature of normal stem cells. As these cells developed, RARγ expression and expression of RARα and RARβ increased [69]. Expression of the RARγ paralog is restricted to stem and primitive progenitor cells during zebrafish embryonic development [70]. Attribution of the above characteristics to patients’ cancer cells might reflect a prevalence of cancer stem/stem-like cells. Leukemia and cancer stem cells are rare cells (<0.1%) as measured by cells that give rise to disease when transplanted into NOD/SCID mice, for example, for acute myeloid leukemia [13]. The use of a more immune-compromised mouse, namely NOD/SCID interleukin-2 receptor gamma chain null, for transplantation, showed that 27% of patients’ melanoma cells gave rise to a tumor from single cell transplants [71]. The detection of cancer stem cells was substantially increased because only 0.0001% of human melanoma cells gave rise to tumors in NOD/SCID mice [72]. Hence, the frequencies of cancer stem cells for many cancers may be an underestimate, and there is a need to measure the levels of ATRA and RARγ within cancer stem cells versus their normal stem cell counterparts. Even so, bulk patients’ cells were analyzed in studies, and it seems likely that low ATRA synthesis, RARγ overexpression, and low miR-30a-5p expression pertain to most, if not all, of the cells. Moreover, if these attributes do reflect a prevalence of stem cell-like cells, they are by no means behaving as normal stem cells (see above).

## 6. The Consequences of Active and Inactive RARγ

RARγ preferentially regulates the behavior of normal stem cells. Transplantable cells in the bone marrow of femurs were reduced in RARγ knockout mice and, therefore, RARγ expression, and when either active or inactive, was important to the maintenance and/or self-renewal of hematopoietic stem cells [69]. Treatment of zebrafish embryos at 4 h post-fertilization with a selective RARγ agonist, in the absence of ATRA for RARα activation, disrupted stem cell decision-making for the patterning of zebrafish development, as seen from tissue disorganization and the failure of some tissues to develop [73]. In this case, active RARγ exerted a negative regulatory role regarding the onset of stem cell differentiation and/or the choice of lineage fate. From studies of embryonic stem cells, active RARγ2 has been proposed to exert either a positive influence on neuronal cell differentiation or a negative influence to eliminate other fates [74]. 

Overexpression of RARγ is associated with cancer cell proliferation. Similarly, treatment of the prostate cancer cell lines DU-145, LNCaP, and PC3 with the RARγ agonist AGN205327, or a low level of ATRA to activate just RARγ, stimulated cell growth and colony formation by the cancer stem cell-like cells. Treatment of the prostate cancer cell lines with the RARγ antagonist AGN205728 and the pan-RAR antagonist AGN194310 led to growth arrest in the G1 phase of cell cycle followed by necroptosis. This was mitochondrial-dependent, caspase-independent, and blocked by inhibition of the poly (ADP-ribose) polymerase PARP-1. Colony formation by the cell line cells was prevented by the RAR antagonists [75,76]. Both antagonists were very effective against patients’ prostate cancer cells, and the pan-RAR antagonist ablated neurosphere formation by the cancer stem cells of pediatric patients’ primitive neuroectodermal and astrocytoma tumors and killed the cancer stem cells’ progeny [76]. The RARγ antagonists LY2955303 and MM11253 arrested the proliferation of the Panc-1 and PK-1 pancreatic cancer cell lines in the G1 phase of cell cycle without causing apoptosis, and the use of patient-derived organoids confirmed a tumor suppressive effect [43]. The natural flavonoid acacetin displaced [^3^H]ATRA from the ligand binding domain of RARγ and targets the non-genomic actions of RARγ (see below). It has activity against lung, breast, and prostate cancer cells, and strongly inhibited the growth of and induced apoptosis of hepatocellular cancer cells. Cell death was primarily attributed to apoptosis because acacetin induced PARP-1 cleavage in some lines but not others [77]. 

Targeting to prevent endogenous ATRA synthesis has included the use of 673A, a DEAB analogue which inhibits ALDH1A isoforms; DIMATE, an irreversible inhibitor of ALDH1 and ALDH3; NCT-501, a theophylline-based specific inhibitor of ALDH1A1; silybin (HY-13748), an inhibitor of ALDH1A1 expression; and solomargine, to downregulate the expression of ALDH1 isoforms. Preventing ATRA synthesis reduced the survival of cancer cells. Furthermore, 673A induced necroptosis of CD133+ ovarian cancer stem-like cells, which was mediated by the induction of mitochondrial uncoupling proteins and reduced oxidative phosphorylation [78]. DIMATE was effective against small-cell lung cancer cell xenografts and elicited oxidative stress-mediated apoptosis of treated H1650 and H1975 cell line cells [79]. DEAB, NCT-501, and disulfiram, an ALDH inhibitor, reduced spheroid cell formation by patients’ uterine endothelial cancer stem cells and caused cell death preferentially in ALDH-high cells. Disulfiram suppressed tumorigenesis of spheroid cells in vivo [80]. Silybin reduced tumor growth when ALDH1A1+ prostate cancer DU145 cells were transplanted into nude mice. Intriguingly, silybin downregulated the expression of RARα [81]. Solomargine decreased the viability of ovarian cancer cell line cells and inhibited the growth of the human ovarian cell line A2780CP70 in mouse xenografts [82]. 

Table 2 summarizes the agents that have been used to interfere with ATRA signaling within cancer cells, their targets, and the cancer cells that the agents are effective against.

From all the above, RARγ influences the survivability of stem cells and whether they self-renew or differentiate, including the availability of lineage options. These multiple influences are all germane to understanding how RARγ overexpression deregulates the behavior of cancer stem cells. 

## 7. The Modes of Action of RARγ

RARγ was overexpressed in the cytoplasm of cancer cells for reasons that are unknown, e.g., hepatocellular cancer [40], or within the nucleus, e.g., pancreatic cancer [43]. Proposed modes of cytoplasmic action include an influence on intracellular signal transduction pathways that control cell survival, proliferation, and differentiation. RARγ within the nucleus regulates many genes allowing ATRA to regulate various gene expression programs to different aspects of cell behavior.

A role in modulating intracellular signaling for cell survival and growth was proposed for RARγ overexpression in the cytoplasm of hepatocellular cancer cells. For these cells, coimmunoprecipitation of RARγ and p85a was observed, and p85α is the regulatory subunit of phosphoinositide 3-kinase, a key regulator of cell survival and proliferation. The oncogenic activity of RARγ was attributed to interaction with p85a, leading to the constitutive activation of AKT and NF-kB and the promotion of cell survival and growth [40]. As mentioned above, acacetin targeting of cytoplasmic RARγ within hepatocellular carcinoma cells led to apoptosis, which was attributed to AKT-p53 switching from being pro-survival to pro-apoptotic [77]. Prostate cancer cells underwent necroptosis when treated with the RARγ antagonist (see above). The ripoptosome (Ser/Thr kinases Receptor Interacting Kinase 1(RIPK1)/Receptor Interacting Kinase 3 (RIPK3)) death complex drives necroptosis and RARγ is required for its formation. When mouse embryonic fibroblasts were treated with DNA-damaging compounds, RARγ was released from the nucleus to the cytosol and complexed with RIPK1 but was not present in the final complex. These studies revealed that cytoplasmic RARγ regulates DNA damage-induced cell death [83]. The presence of RARγ within the cytoplasm can promote cell differentiation. Co-immunoprecipitation and biophysical studies using neuroblastoma cell lines revealed binding of RARγ to c-Src. This was ligand-dependent and resulted in c-Src activation, which was required for neuritogenesis [84]. 

RARγ within the nucleus regulates an astonishingly large network of genes as revealed from integrative genomic studies of F9 embryonal stem cells. Post ATRA-treatment, 281 and 926 genes were induced (≥1.8-fold) by 2 and 48 h, respectively, resulting from RARγ/RXR co-occupancy [85]. RARγ regulates stem cell pluripotency/self-renewal because the binding sites for RAR/RXR dimers within undifferentiated F9 embryonal stem cells coincided with loci that are targeted by the transcription factors SOX2, NANOG, and POU5F1, which are important to stem cell stemness, and RAR/RXR dimers can distinguish pluripotency- and differentiation-associated cis-regulatory elements [86]. For ATRA-treated embryonic stem cells, RARγ was essential for gene expression by virtue of regulating chromatin marks for remodeling. When RARγ^−/−^ cells were compared to the wild-type cells, the levels of 80 transcripts were reduced and the levels of 78 transcripts were increased. From these findings, the investigators suggested a role for RARγ in regulating gene expression when ligand was absent [45]. As mentioned above, RARγ-mediated chromatin epigenetic activation was also seen for pancreatic cancer cells. From studies of embryonic stem cell differentiation, RARγ was required for the broad epigenetic organization of the *Hoxa* and *Hoxb* gene clusters and the gene-specific removal of the polycomb-repressive mark H3K27me3. Activation of the gene cluster was attenuated by the deletion of the RARγ binding site within the *Hoxa1* enhancer [87]. Treatment of lung cancer stem cells with the synthetic retinoid WYC-209 revealed a role for RARγ in regulating the overall status of chromatin. Post-treatment, translocation of RARγ from the nucleus to the cytoplasm reduced RARγ binding to the promotor region of the *cell division control 42 (Cdc42)* gene. Down-regulation of Cdc42 protein expression, which regulates actin polymerization, reduced filamentous actin, which inhibited cytoskeletal tension resulting in chromatin de-condensation [88].

How overexpression of RARγ deregulates the survivability, proliferation, and/or differentiation, including the availability of lineage options of cancer stem cells, remains to be unraveled, particularly regarding the capacity of RARγ to endow cancer stem cells with an aggressive behavior leading to poor disease prognosis. Adding complexity to unraveling the role(s) of RARγ within the nucleus, different RAR/RXR heterodimers bind at identical targets and there is orchestrated recruitment and release regarding how the expression of repertoires of target genes is regulated [85]. It is important to bear in mind that there is still a paucity of information regarding a direct comparison of RARγ-mediated events within cancer stem cells versus their normal stem cell counterparts.

## 8. Concluding Remarks

Studies have shown that (1) intracellular ATRA is low within various cancer cells, (2) the enzymes required for ATRA synthesis are often absent, (3) RARγ is an oncogene for some cancers and transactivated by nM ATRA, (4) the miR-30a-5p is a tumor suppressor and expression is low in cancers that overexpress RARγ, and (5) the miR-30a-5p is a negative regulator of RARγ expression. Collectively, these findings provide strong support to the notion that perturbations to ATRA signaling play a role in deregulating the behavior of cancer cells. It is not entirely clear how deregulations of ATRA-, RARγ-, and miR-30-5p-mediated events lead to the abnormal behavior in cancer stem cells and their progeny. Regarding the widespread influences of RARγ and miR-30-5p, there is the capacity to change gene expression to many aspects of the control of cancer stem cell behavior, including the hallmark circuits that include for viability, proliferation, differentiation, cytostasis, and motility [89]. As mentioned above, the influence of RARγ on embryonic stem cells is to either promote neural differentiation or eliminate other fates, and agonism of RARγ exerted a negative influence on tissue patterning and the generation of some tissues regarding the early development of zebrafish embryos. It is interesting to speculate whether RARγ plays a role in restricting the fate of cancer stem cells so that they produce an overwhelming abundance of lineage-restricted, unwanted, and antisocial cells. 

For some years, investigators have looked to target therapies to cancer stem cells that are quiescent because they are resistant to conventional chemotherapeutics [90,91]. This need is pressing because the COVID-19 pandemic disruption to cancer diagnosis and treatment will lead to people dying for several years to come from otherwise manageable cancers. Targeting RARγ, by the AGN205728 and LY2955303 antagonists and acacetin, and interference with ATRA synthesis, by 637A, DIMATE, NCT-501, silybin, and solomargine, were effective against cancer stem cells. The RARγ antagonist and 637A induced necroptosis, a failsafe cell death mechanism, which is important because some cancer cells are resistant to undergoing apoptosis. Whilst there is still some uncertainty regarding the status of ATRA synthesis and signaling within normal tissue-specific stem cells, there is a difference between the sensitivity of cancer cells and normal cells to agents that target RARγ and prevent ATRA synthesis. The RARγ antagonist AGN205728 was more effective against prostate cancer cells than normal prostate epithelium and normal prostate RWPE-1 cells [76] and did not affect cultures of human hematopoietic stem cells [37]. Acacetin was effective against hepatocellular and colorectal cancer cell lines, and normal liver cells were resistant [77]. In addition, 673A, an ALDH1A, inhibitor showed selectivity for ovarian cancer stem cells over normal cells and little toxicity to human mesenchymal stem cells and non-malignant MCF-10A breast cells [78]. DIMATE, an ALDH1 and ALDH3 inhibitor, eradicated leukemia stem cells and spared normal hematopoietic progenitors [92]. The use of a pan-RAR antagonist in rodents appears to be safe. Other than an inhibition of spermatogenesis, which was reversible, no adverse effects were seen when mice and rats were given substantial doses of the pan-RAR antagonist BMS-18945 [93,94]. Targeting ATRA signaling is highly promising as a means to manage, perhaps even cure, cancer stem cell-mediated aggressive disease and cancer relapse.

## Figures and Tables

**Table 1:** ATRA synthesis and signaling in cancer.

Cancer Type	ATRA Synthesis	RARγ is an Oncogene	miR-30a-5p is a Tumor Suppressor
Breast	No/low ATRA synthesis by human cell lines [27,28]		Expression in human cell lines inhibited proliferation [61]
Colorectal	ADH1B, ADH1C, RDHL, RDH5 mRNAs low in patients’ cells and human cell lines [19] RDHL and RDH5 mRNAs low in patients’ cells and human cell lines, low ATRA synthesis by cell lines [20]	Overexpressed in patients’ cells and human cell lines [38]	Low in patients’ cells, suppression in a xenograft model [52]
Cholangiocarcinoma		Overexpressed in patients’ cells, downregulation reduced tumor xenografts [39]	Tumor promoter, overexpression promoted HCC9810 proliferation [66]
Gastric	ADH4, ADH1B, ADH1C, RDHL, ALDH1A1 mRNAs low in patients’ cells [21]		Low in patients’ cells, linked to metastasis [53]
Hepatocellular		Overexpressed in patients’ cells, overexpression in HepG2 promoted xenograft growth [40]	Low in patients’ cells, expression in a human cell line inhibited proliferation [54]
Lung	ADH1B, ADH3, RDH1, ALDH1A1 mRNAs low in patients’ cells [23]		Low in patients’ cells, a marker of disease progression [55]
Oropharyngeal	ALDH1A2 expression low in patients’ cells [24]		Low in patients’ cells, expression in human cell lines inhibited proliferation [56]
Ovarian	No ATRA synthesis by human cell lines [29]; ALDH1A2, ALDH1B1, ALDH9A1 mRNAs low in human cell lines [30]	Overexpressed in patients’ cells, knockdown in A2780 reduced xenograft growth [41]	Low in human cell lines, expression inhibited proliferation [64]
Pancreatic	Low ATRA in patients’ cells [31]	Overexpressed in patients’ cells, required for the proliferation of pancreatic cancer cells [42,43]	Low in patients’ cells, suppression in a xenograft model [57]
Prostate	Low ATRA within patients’ cells [33]		Low in patients’ cells, expression inhibited human cell line proliferation [58]
Renal	Low all-*trans* retinol and retinyl esters within patients’ cells [34]	Overexpression in patients’ cells [44]	Low in patients’ cells, expression inhibited human cell line proliferation [59]

**Table 2:** Targeting ATRA signaling to kill cancer cells.

Agent	Target	Activity against
AGN205728	RARγ antagonist	Patients’ prostate cancer cells, human cell lines [75,76]
AGN194310	Pan-RAR antagonist	Patients’ prostate cancer cells, human cell lines, pediatric patients’ primitive neuroectodermal and astrocytoma tumors [76]
LY2955303 MM11253	RARγ antagonists	Panc-1 and PK-1 pancreatic cancer cell lines, patient-derived organoids [43]
Acacetin	Non-genomic actions of RARγ	Human hepatocellular cancer cell lines [77]
673A	Inhibits ALDH1A isoforms	Human CD133+ ovarian cancer stem-like cells [78]
DIMATE	Inhibits ALDH1 and ALDH3	Small cell lung cancer cell xenografts, human cancer cell lines [79]
DEAB, NCT-501, disulfiram	Inhibits ALDH1A1	Spheroid cell formation by patients’ uterine endothelial cancer stem cells, tumorigenesis in vivo (disulphiram) [80]
Silybin (HY-13748)	Inhibits ALDH1A1 expression	Xenografts of DU145 prostate cancer cell line cells [81]
Solomargine	Downregulates ALDH1 isoforms expression	Xenografts of A2780CP70 ovarian cancer cell line cells, human cell lines [82]

## Data Availability

Not applicable.
